# 5-HT_2C_R blockade in the amygdala conveys analgesic efficacy to SSRIs in a rat model of arthritis pain

**DOI:** 10.1186/1744-8069-9-41

**Published:** 2013-08-12

**Authors:** Stéphanie Grégoire, Volker Neugebauer

**Affiliations:** 1 Department of Neuroscience and Cell Biology, The University of Texas Medical Branch, Galveston Texas 77555-1069, USA

**Keywords:** Amygdala, Pain, Serotonin, SSRI, 5-HT_2C_R, Emotional-affective behavior, Anxiety

## Abstract

**Background:**

Pain, including arthritic pain, has a negative affective component and is often associated with anxiety and depression. However, selective serotonin reuptake inhibitor antidepressants (SSRIs) show limited effectiveness in pain. The amygdala plays a key role in the emotional-affective component of pain, pain modulation and affective disorders. Neuroplasticity in the basolateral and central amygdala (BLA and CeA, respectively) correlate positively with pain behaviors. Evidence suggests that serotonin receptor subtype 5-HT_2C_R in the amygdala contributes critically to anxiogenic behavior and anxiety disorders. In this study, we tested the hypothesis that 5-HT_2C_R in the amygdala accounts for the limited effectiveness of SSRIs in reducing pain behaviors and that 5-HT_2C_R blockade in the amygdala renders SSRIs effective.

**Results:**

Nocifensive reflexes, vocalizations and anxiety-like behavior were measured in adult male Sprague–Dawley rats. Behavioral experiments were done in sham controls and in rats with arthritis induced by kaolin/carrageenan injections into one knee joint. Rats received a systemic (i.p.) administration of an SSRI (fluvoxamine, 30 mg/kg) or vehicle (sterile saline) and stereotaxic application of a selective 5-HT_2C_R antagonist (SB242084, 10 μM) or vehicle (ACSF) into BLA or CeA by microdialysis. Compared to shams, arthritic rats showed decreased hindlimb withdrawal thresholds (increased reflexes), increased duration of audible and ultrasonic vocalizations, and decreased open-arm choices in the elevated plus maze test suggesting anxiety-like behavior. Fluvoxamine (i.p.) or SB242084 (intra-BLA) alone had no significant effect, but their combination inhibited the pain-related increase of vocalizations and anxiety-like behavior without affecting spinal reflexes. SB242084 applied into the CeA in combination with systemic fluvoxamine had no effect on vocalizations and spinal reflexes.

**Conclusions:**

The data suggest that 5-HT_2C_R in the amygdala, especially in the BLA, limits the effectiveness of SSRIs to inhibit pain-related emotional-affective behaviors.

## Background

Pain is a multidimensional experience that includes not only sensory-discriminative but also emotional-affective and cognitive components [1,2]. Certain antidepressants have become part of the therapeutic strategy for different types of persistent pain, including neuropathic pain, fibromyalgia, low back pain and headache [3-6], and they are also considered for osteoarthritis pain [7]. Selective serotonin reuptake inhibitor antidepressants (SSRIs) have low or inconsistent analgesic efficacy [4,6] but better overall safety and tolerability compared to tricyclic antidepressants [8].

The serotonergic system has long been known to play an important role in pain modulation [9,10]. The family of at least 14 serotonin (5-HT) receptor subtypes is divided into seven groups (5-HT_1_R – 5-HT_7_R) based on their structural and functional characteristics [11-13]. The heterogeneity of 5-HT receptors is believed to account for the differential inhibitory or excitatory effects of 5-HT in the descending pain modulatory systems [9]. 5-HT_2C_ receptor (5-HT_2C_R) has emerged as a major target for improved treatment of neuropsychiatric disorders such as anxiety disorders [14-16]. 5-HT_2C_R has also been implicated in adverse effects of 5-HT and SSRIs [14] and in inconsistent clinical efficacy of SSRIs in neuropathic pain [17]. 5-HT_2C_R is a G_q/11_ protein-coupled receptor expressed in GABAergic, glutamatergic, and dopaminergic neurons [18,19]. Thus, 5-HT_2C_R can regulate the release of different transmitters to modulate excitatory and inhibitory neurotransmission [20-22]. 5-HT_2C_R mRNA and protein show widespread distribution in the human and rat brain, including in the amygdala where particularly high levels are found in the lateral-basolateral area [23,24].

The amygdala, a subcortical area known for its key role in emotions and affective disorders [25], is now recognized as an important neural substrate for the emotional-affective dimension of pain based on preclinical studies from our group [for reviews see [26,27]] and others [28-31] and clinical work [32,33]. Amygdala activity correlates positively with pain behaviors in animals. Increases of amygdala activity can elicit or enhance pain responses even in the absence of tissue injury [34-41]. Conversely, deactivation of the amygdala inhibits pain in different animal models [28,42-49]. Recent studies in humans also show increased amygdala activity in experimental and clinical pain [50-54]. The amygdala circuitry that contributes to emotional-affective aspects of pain is centered on the lateral-basolateral (LA-BLA) and central (CeA) nuclei [26,27]. The CeA receives nociceptive information through a direct pathway from the spinal cord and brainstem (external lateral parabrachial area) and highly processed affect-related information through an indirect pathway from the LA-BLA network via posterior thalamus [26,27]. Neuroplasticity characterized by enhanced excitatory transmission [44,45,55-61] and loss of inhibitory control [61,62] develops in this circuitry in models of inflammatory and neuropathic pain. As a result, abnormally enhanced CeA output generates emotional-affective behaviors and modulates nocifensive responses through direct and indirect projections to brainstem and forebrain areas [26,27].

The amygdala receives a strong serotonergic projection from the dorsal raphe nucleus [63,64], which exerts excitatory and inhibitory effects on neuronal activity through different receptor subtypes [65,66]. There is evidence for increased 5-HT release in the amygdala (BLA) in aversive states [16,67,68]. 5-HT_2C_R in the BLA but not CeA contributes critically to anxiogenic behavior and anxiety disorders [15,16,69] and mediates anxiogenic side effects of acutely administered antidepressants such as SSRIs [4,70,71]. Synaptic and cellular effects of 5-HT_2C_R in the amygdala are largely unknown but 5-HT_2C_R activation in the BLA facilitated NMDA receptor-mediated synaptic plasticity in BLA neurons [72] and induction of hippocampal LTP [73], suggesting that 5-HT_2C_R can control amygdala output.

In this study, we tested the hypothesis that 5-HT_2C_R in the amygdala (BLA but not CeA) contributes to the limited effectiveness of SSRIs on pain behaviors and that blockade of 5-HT_2C_R in the BLA renders SSRIs effective in reducing emotional-affective behaviors in a model of arthritis pain. To do so we applied a selective 5-HT_2C_R antagonist (SB242084 [74]) stereotaxically into BLA or CeA and administered a selective SSRI (fluvoxamine [75]) systemically (intraperitoneally, i.p.). The effect of each drug alone and of their combined application was determined.

## Results

**Figure 1:**
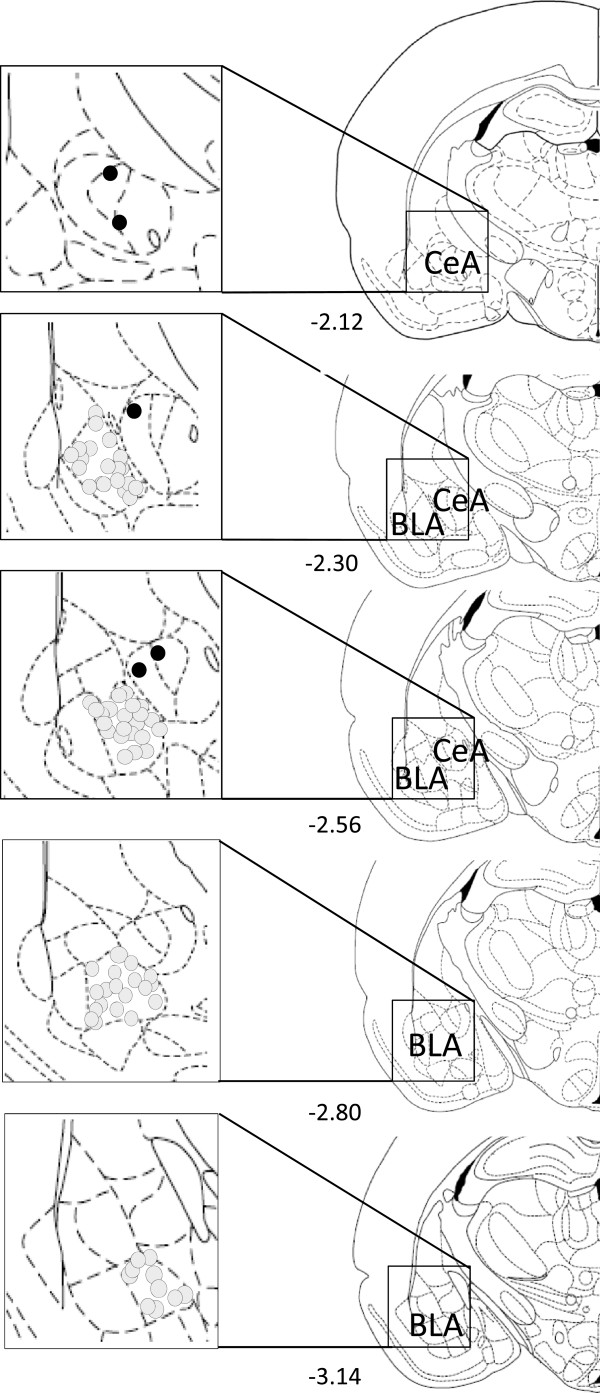
**Histological verification of drug application sites.** Diagrams adapted from Paxinos and Watson [107] show coronal sections through the right hemisphere at different levels posterior to bregma (indicated by numbers). Next to each diagram are shown in detail the basolateral (BLA) and central nuclei (CeA) of the amygdala. The boundaries of the different amygdala nuclei are easily identified under the microscope (see Figure 1 in [45]). Each symbol indicates the location of the tip of one microdialysis probe. Open circles, BLA; filled circles, CeA.

Spinal reflexes (hindlimb withdrawal thresholds), audible and ultrasonic vocalizations, and anxiety-like behavior in the elevated plus maze (EPM) were measured in adult male Sprague Dawley rats with (n = 61) or without (n = 38) arthritis. Arthritic pain was induced by intraarticular injections of kaolin and carrageen into the left knee joint. Sham rats were handled the same way as arthritic rats but the needle was inserted into the knee without injecting any compounds (see Methods). Behaviors and drug effects were measured 5–6 h after arthritis induction or needle insertion (shams). Drugs or vehicle were administered systemically (i.p.) and stereotaxically into the right basolateral (BLA) or central (CeA) nuclei of the amygdala (see Figure 1). Each animal was tested with only one drug regimen. Audible and ultrasonic vocalizations were measured simultaneously (see Methods) and spinal reflex thresholds were also determined in these animals (counter-balanced for order of tests). EPM performance was tested in separate groups of animals.

### Co-application of intra-BLA SB242084 and systemic fluvoxamine decreased vocalizations and anxiety-like behavior of arthritic rats

#### Audible vocalizations

**Figure 2:**
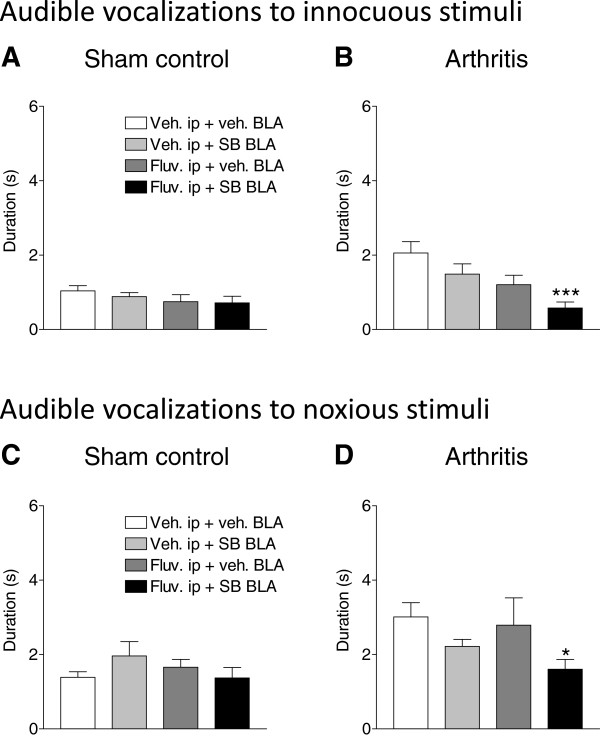
**Drug effects on audible vocalizations.** Effects of intra-BLA infusion of a selective 5-HT_2C_R antagonist (SB242084, 10 μM), systemic administration of an SSRI (fluvoxamine, 30 mg/kg, i.p) and combined administration of SB242084 and fluvoxamine on audible vocalizations evoked by innocuous (300 g/30 mm^2^, **A,B**) and noxious (1200 g/30 mm^2^, **C,D**) stimulation of the knee joint in sham control **(A,C)** and arthritic **(B,D)** rats. The drug combination had significant inhibitory effects in the arthritis pain model. *,*** P < 0.05, 0.001, compared to vehicle control (first bar in each histogram), one-way ANOVA followed by Dunnett’s multiple comparison tests. Bar histograms show means ± SEM of the total duration vocalizations over a 1 min period following the onset of the mechanical stimulus.

Audible vocalizations evoked by innocuous (300 g/30 mm^2^) and noxious (1200 g/30 mm^2^) compression of the knee for 15 s were measured in sham controls (Figure 2A and 2C) and in arthritic rats (Figure 2B and 2D). In sham rats, the following drug regimen had no effect on the duration of audible vocalizations compared to vehicle (n = 6 rats): stereotaxic application of a selective 5-HT_2C_R antagonist (SB242084 [74], 10 μM, concentration in microdialysis fiber) into BLA together with systemic vehicle administration (n = 7 rats); systemic administration of a selective SSRI (fluvoxamine [75], 30 mg/kg, i.p.) together with ACSF vehicle application into BLA (n = 7 rats); and combined application of systemic fluvoxamine and intra-BLA SB242084 (n = 7 rats). Arthritic rats showed increased audible vocalizations to normally innocuous (Figure 2B) and noxious (Figure 2D) stimuli, reflecting allodynia and hyperalgesia, respectively (see vehicle-treated group, n = 7 rats). Intra-BLA application of SB242084 (10 μM, n = 6) or systemic administration of fluvoxamine (30 mg/kg, i.p., n = 5) had no effect on audible vocalizations of arthritic rats. However, the combination of intra-BLA SB242084 and systemic fluvoxamine (n = 7) significantly decreased the duration of audible vocalizations to innocuous (P < 0.001) and noxious stimuli (P < 0.01, Dunnett’s multiple comparison tests), reversing the effect of arthritis. The data suggest that 5-HT_2C_R blockade allows an SSRI to inhibit higher integrated pain behaviors.

#### Ultrasonic vocalizations

**Figure 3:**
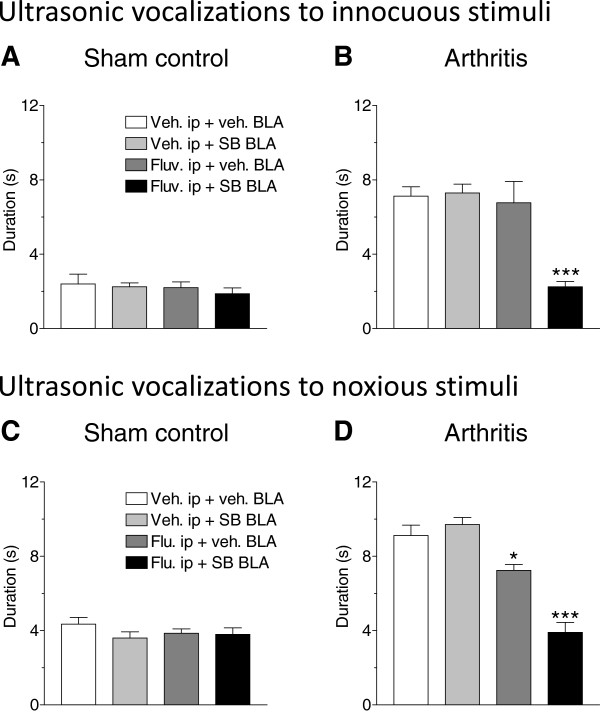
**Drug effects on ultrasonic vocalizations.** Effects of intra-BLA infusion of the selective 5-HT_2C_R antagonist (SB242084, 10 μM), systemic administration of the SSRI fluvoxamine (30 mg/kg, i.p) and combined administration of SB242084 and fluvoxamine on audible vocalizations evoked by innocuous (300 g/30 mm^2^, **A,B**) and noxious (1200 g/30 mm^2^, **C,D**) stimulation of the knee joint in sham control **(A,C)** and arthritic **(B,D)** rats. The drug combination had significant effects in the arthritis pain state. *,*** P < 0.05, 0.001, compared to vehicle control, one-way ANOVA followed by Dunnett’s multiple comparison tests. Same display as in Figure 2.

Ultrasonic vocalizations evoked by innocuous (300 g/30 mm^2^) and noxious (1200 g/30 mm^2^) stimulation of the knee joint were measured in sham control (Figure 3A and 3C) and arthritic rats (Figure 3B and 3D). In sham rats, none of the drug regimen (SB242084, 10 μM, n = 7; fluvoxamine, 30 mg/kg, n = 7; co-application of SB242084 and fluvoxamine, n = 7 rats) had a significant effect on the duration of ultrasonic vocalizations compared to vehicle (n = 6 rats). Arthritic rats showed increased vocalizations to normally innocuous (Figure 3B) and noxious (Figure 3D) stimuli (see vehicle-treated group, n = 7 rats). The combination of intra-BLA SB242084 and systemic fluvoxamine (n = 7) inhibited ultrasonic vocalizations significantly (P < 0.001, Dunnett’s multiple comparison tests) compared to vehicle (n = 7 rats). SB242084 alone had no effect (n = 6) and fluvoxamine alone inhibited ultrasonic vocalizations to noxious stimuli slightly but significantly (n = 5, P < 0.05). The data suggest that 5-HT_2C_R blockade induces or enhances the ability of an SSRI to inhibit affective pain behaviors.

#### Anxiety-like behavior and locomotion

**Figure 4:**
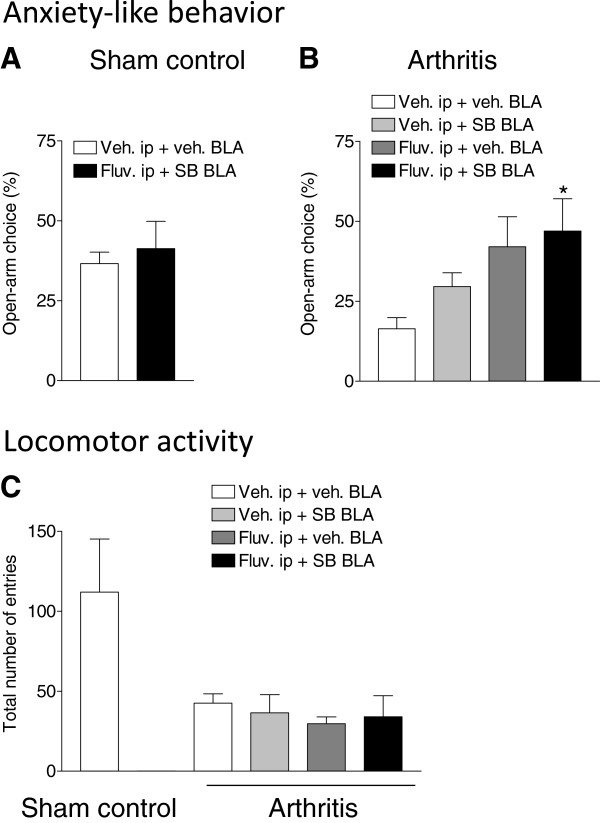
**Drug effects on anxiety-like behavior and locomotion.** Open-arm choice in the elevated plus maze (EPM) was measured for 5 min as the ratio of open-arm entries to the total number of entries expressed as % in sham controls **(A)** and arthritic animals **(B)**. The combination of intra-BLA SB242084 (10 μM) and systemic fluvoxamine (30 mg/kg) had no effect in sham controls but increased the open-arm choice of arthritic rats significantly. *P < 0.05, compared to vehicle, one-way ANOVA followed by Dunnett’s multiple comparison tests). **(C)** Evaluation of locomotor activity (total number of entries into the 4 arms of the EPM) in sham controls (left bar) and arthritic animals for 30 min. Locomotor activity was decreased in arthritic rats. The different drug regimen tested in arthritic rats had no effect. Bar histograms show means ± SEM.

Open-arm choice in the elevated plus maze (EPM) was measured for 5 min in sham control rats and in arthritic rats as a negative indicator of anxiety-like behavior [76]. Compared to sham controls (n = 6), arthritic animals (n = 6) showed decreased preference for the open arms indicating an increase in anxiety-like behavior in the pain state (see vehicle control groups in Figure 4A and 4B). The combination of intra-BLA SB242084 (10 μM) and systemic fluvoxamine (30 mg/kg) had no effect in control rats (n = 5) compared to vehicle (n = 6) but increased the open-arm choice of arthritic rats (n = 6) compared to vehicle (n = 6) significantly (P < 0.05, Dunnett’s multiple comparison tests), suggesting an anxiolytic effect in the pain state. Intra-BLA SB242084 (n = 7) or systemic fluvoxamine (n = 7) alone had no significant effect in arthritic rats. Compared to sham controls (n = 6), arthritic rats (n = 6) showed decreased exploratory behaviour measured as the total number of entries into the open and closed arms of the EPM for 30 min (Figure 4C). None of the drug regimen (SB242084, 10 μM, n = 7; fluvoxamine, 30 mg/kg, n = 7; co-application of SB242084 and fluvoxamine, n = 6 rats) had a significant effect on locomotor activity in arthritic rats. In sham controls, only the combination of SB242084 and fluvoxamine was tested and had no significant effect on locomotor activity (n = 5).

### Co-application of intra-BLA SB242084 and systemic fluvoxamine had no effect on spinal reflexes

**Figure 5:**
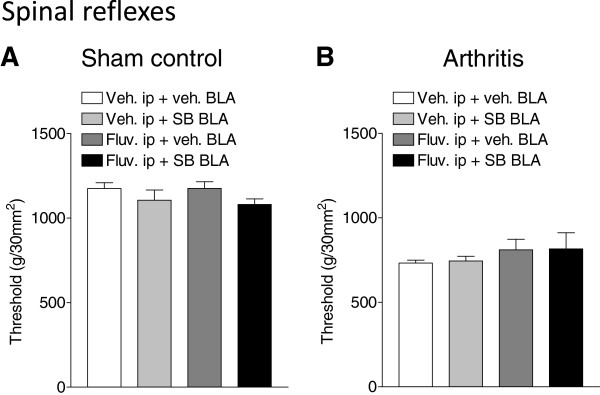
**Drug effects on hindlimb withdrawal thresholds.** Reflex thresholds were measured in sham controls **(A)** and arthritic rats **(B)** using a calibrated forceps for mechanical compression of the knee joint. Intra-BLA application of SB242084 (10 μM), systemic administration of fluvoxamine (30 mg/kg, i.p), and combined administration of SB242084 and fluvoxamine had no effect on reflex thresholds in control or arthritic rats. Bar histograms show means ± SEM.

Thresholds of hindlimb withdrawal reflexes evoked by mechanical compression of the knee joint were measured in sham controls (Figure 5A) and arthritic rats (Figure 5B). Compared to controls (n = 6 rats) arthritic rats (n = 7) had decreased thresholds, reflecting increased spinally organized reflexes and mechanical hypersensitivity (see vehicle groups in Figure 5A and 5B). None of the drug regimen had a significant effect in control rats (SB242084, 10 μM, n = 7; fluvoxamine, 30 mg/kg, n = 7; co-application of SB242084 and fluvoxamine, n = 7 rats) and in arthritic rats (SB242084, n = 6; fluvoxamine, n = 5; co-application of SB242084 and fluvoxamine, n = 7 rats). The data suggest that blockade of 5-HT_2C_R in the BLA allowed the serotonergic system to affect supraspinally but not spinally organized behaviors.

### Co-application of intra-CeA SB 242084 and systemic fluvoxamine had no effect on spinal reflexes and vocalizations in arthritic rats

**Figure 6:**
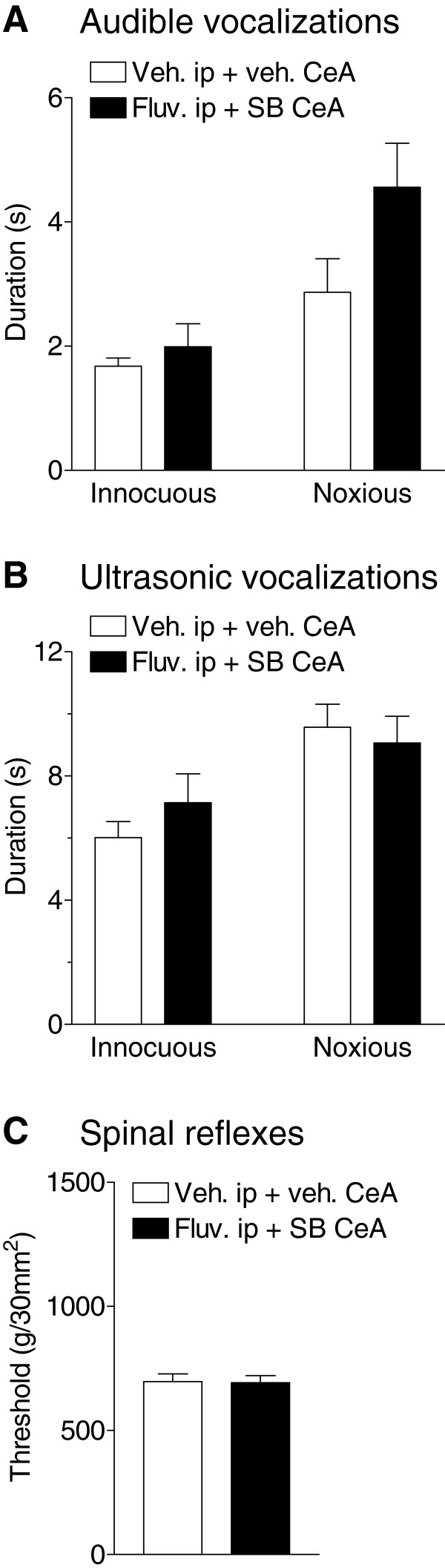
**Drug application into CeA.** Audible **(A)** and ultrasonic **(B)** vocalizations to innocuous and noxious compression of the knee (see Figure 2) and hindlimb withdrawal thresholds (**C**, see Figure 5) were measured in arthritic rats 5–6 h postinduction. Compared to vehicle controls the application of SB242084 (10 μM) into the CeA in combination with systemic administration of fluvoxamine (30 mg/kg, i.p) had no significant effect. Bar histograms show means ± SEM.

Since the CeA serves as a major output nucleus for amygdala function related to pain modulation, we tested if 5-HT_2C_R also played a role in this nucleus (Figure 6). We only tested the combination of SB242084 and fluvoxamine since the previous results showed that fluvoxamine alone and SB242084 applied into BLA had no effect. We also performed these tests in arthritic animals only since the combination of intra-BLA SB242084 and systemic fluvoxamine had no effect in normal animals. The results show that coapplication of intra-CeA SB242084 (10 μM) and systemic fluvoxamine (30 mg/kg) had no significant effect on audible and ultrasonic vocalizations to (normally) innocuous and noxious stimuli and on spinal reflexes in arthritic rats (n = 5 rats for each parameter).

## Discussion

The novelty of this study is the finding that blockade of 5-HT_2C_R in the BLA, but not CeA, can induce or enhance the ability of an SSRI to inhibit emotional-affective pain behaviors in an animal model of arthritis pain. The results suggest that 5-HT_2C_R in the BLA prohibits beneficial pain-inhibiting effects of serotonin (5-HT), which is consistent with previous reports that 5-HT_2C_R in the amygdala (BLA but not CeA) contributes critically to anxiogenic behavior and anxiety disorders [16,69,77] and mediates anxiogenic side effects of antidepressants including SSRIs [4,70,78]. 5-HT_2C_R antagonists have anxiolytic and antidepressant effects [79-82], block the SSRI-induced increase of fear expression [71] and potentiate the antidepressant effects of SSRIs [83].

The present study provides not only further evidence for an important role of the amygdala in emotional-affective aspects of pain [26,27] but also identifies the serotonergic system as a powerful modulator of amygdala function in pain. Blockade of 5-HT_2C_R by itself had no effect, but increasing the serotonergic drive with an SSRI engaged this receptor so that its blockade allowed 5-HT to inhibit pain behaviors. Since this strategy affected supraspinally organized behaviors (vocalizations and anxiety-like behaviors) but not spinal reflexes, the beneficial 5-HT effects appear to be due to an action in the brain. Our data suggest that 5-HT_2C_R acts in the BLA to block pain-inhibiting effects of SSRIs. The synaptic and cellular mechanisms remain to be determined.

The amygdala receives serotonergic input predominantly from the dorsal raphe nucleus [63,64] and increased 5-HT release in the amygdala (BLA) is associated with aversive states [16,67,68]. 5-HT_2C_R activation in the BLA increased synaptic activation of BLA neurons [72]. A possibly scenario to explain the findings of our study is that 5-HT_2C_R activation drives BLA output to increase activity in the CeA, which serves as the output nucleus for major amygdala function and accounts for amygdala-dependent emotional-affective aspects of pain [26,27]. 5-HT_2C_R knockout data linked 5-HT_2C_R-mediated anxiogenic behavior to the activation of CRF-containing neurons in the CeA [15]. Our previous studies established an important role of the CRF system in pain-related amygdala functions [35,45,84-86]. CeA neurons project directly or indirectly to brainstem and forebrain areas involved in the expression of aversive behaviors and pain modulation, including the periaqueductal gray [26,87-89]. Direct brainstem projections from CeA can be glutamatergic [90], but CRF-containing CeA neurons also include a population of GABAergic neurons [91,92]. Therefore, the positive correlation between amygdala output and pain behaviors can result from descending facilitation or disinhibition.

A single administration of fluvoxamine had pain-relieving effects in our study when combined with blockade of 5-HT_2C_R in the BLA, which is consistent with the observation that analgesic effects of anti-depressants are independent of their anti-depressant effects that usually occur only after weeks [5,7]. Fluvoxamine alone had no effect except for a slight inhibition of ultrasonic vocalizations evoked by noxious stimulation in arthritic rats. Other studies reported weak effects of systemic fluvoxamine in the formalin pain test [93,94] and mixed effects in the hot plate test [93,95]. Intrathecal fluvoxamine had anti-allodynic effects in a neuropathic pain model (partial nerve ligation) and these were reduced by intrathecal administration of a 5-HT_2A/2C_R antagonist [96]. There is some evidence, however, that 5-HT_2C_R activation in the spinal cord may have inhibitory effects in neuropathic pain models [97,98], possibly mediated by indirect noradrenergic mechanisms [99]. Our data suggest that 5-HT_2C_R activation in the amygdala mediates undesirable effects of 5-HT.

Some technical aspects of our study deserve consideration. We used selective compounds at concentrations that are well established in the literature (SB242084 [22,100-102]; fluvoxamine [75,96]). However, while the drug concentration in the microdialysis fiber is known, the dose administered can only be estimated. Comparative data from our previous microdialysis and in vitro studies [45,55,58,84,85,103,104] indicate that the tissue concentration is at least 100 times lower than in the microdialysis probe due to the concentration gradient across the dialysis membrane and diffusion in the tissue. Therefore, drugs were dissolved in ACSF at a concentration 100 times that predicted to be needed. Microdialysis was chosen for drug delivery because it provides steady state drug levels without a volume effect [105]. Spread of drug and site of action need to be considered. Drug application into the CeA had no effect. These placement control experiments suggest that the drug did not spread beyond a distance of 1 mm around the tip of the microdialysis probe to reach the BLA, which is consistent with our previous estimates [35,48,84,85]. The distance between the tips of the microdialysis probes in the BLA (effective drug administration site) and CeA (ineffective control site) is about 1 mm.

## Conclusion

Pharmacological blockade of 5-HT_2C_R in the amygdala (BLA but not CeA) allows SSRIs to inhibit emotional-affective pain responses and anxiety-like behavior in an arthritis pain model. The study contributes novel insight into 5-HT functions in the brain and into brain mechanisms of pain.

## Methods

### Animals

Male Sprague Dawley rats (225–250 g) were housed in a temperature controlled room and maintained on a 12 h day/night cycle, with free access to food and water. On the day of the experiment, rats were transferred from the animal facility and allowed to acclimate to the laboratory for at least 1 h. At the end of the experiment, the animal was euthanized by decapitation using a guillotine (Harvard Apparatus Decapitator). All experimental procedures were approved by the Institutional Animal Care and Use Committee (IACUC) at the University of Texas Medical Branch (UTMB) and conformed to the guidelines of the International Association for the Study of Pain (IASP) and of the National Institutes of Health (NIH).

### Arthritis pain model and sham controls

A localized mono-arthritis was induced in the left knee joint by intra-articular injections of kaolin (4%, 80–100 μl) and carrageenan (2%, 80–100 μl) through the patellar ligament. This treatment paradigm reliably leads to inflammation and swelling of the knee within 1–3 h, reaches a maximum plateau at 5–6 h, and persists for several days [106]. Therefore, the 5–6 h time point was selected for measuring behaviors and testing drug effects. In sham control rats, the syringe was inserted into the knee joint cavity under the same conditions as in arthritic animals except that no compound was injected. Vehicle was not injected in sham animals because intraarticular saline injection causes a temporary swelling of the joint [55], which is one of the cardinal symptoms of an inflammation. To avoid any latent effect of increased intraarticular pressure, vehicle (sterile saline) was not injected in sham animals.

### Experimental protocol

On Day 1, a guide cannula for drug (or artificial CSF, ACSF) application by microdialysis was stereotaxically inserted into the right BLA or CeA. On Day 2, 5–6 h after the induction of arthritis (or needle insertion for shams), behavioral experiments were performed 30 min after the systemic (i.p.) injection of the fluvoxamine or its vehicle (0.9% NaCl solution) in combination with an intra-BLA or intra-CeA application of SB242084 (or ACSF vehicle) for 20 min. SB242084 (selective 5-HT_2C_R antagonist) and fluvoxamine (SSRI) were purchased from Tocris Bioscience.

### Drug application by microdialysis

Rats were deeply anaesthetized with pentobarbital sodium (Nembutal®, 50 mg/kg, i.p.) on Day 1. A guide cannula (David Kopf Instruments) was stereotaxically implanted into the right BLA or the right CeA, using the following coordinates: BLA, 2.8 mm caudal to bregma, 4.8 mm lateral to midline, 7.6 mm depth; CeA, 2.3 mm caudal to bregma, 4.0 mm lateral to midline, 7.0 mm depth [107]. Guide cannulas were affixed to the skull with dental acrylic (Plastic One, Roanoke, VA). Antibiotic ointment (Solosite Gel, Smith and Nephew) was applied to the exposed tissue to prevent infection. Local application of Lidocaine HCl (1%, 100 μl of 10 mg/ml) was done to minimize surgical pain and to prevent the animal from scratching of the surgical area upon recovery. On Day 2, a microdialysis probe (CMA/Microdialysis 11, Solna Sweden) that extended 1 mm beyond the tip of the guide cannula, was inserted for stereotaxic drug application into the amygdala. The probe was connected to an infusion pump (Harvard Apparatus, Holliston, MA) using polyethylene-50 tubing. Drugs or ACSF (vehicle) were applied for 20 min at a rate of 5 μl/min to establish equilibrium in the tissue. ACSF was oxygenated, equilibrated to pH 7.4 and contained the following (in mM): 125.0 NaCl, 2.6 KCl, 2.5 NaH_2_PO_4_, 1.3 CaCl_2_, 0.9 MgCl_2_, 21.0 NaHCO_3_, and 3.5 glucose. SB242084 was dissolved in ACSF on the day of the experiment at a concentration 100-fold that predicted to be needed in the tissue based on data in the literature [22,100-102] because of the concentration gradient across the dialysis membrane and diffusion in the tissue [45,48,84,85]. At the end of the experiment, rats were decapitated and injection sites were verified histologically after injection of methylene blue (1 μl) and plotted on standard diagrams adapted from Paxinos and Watson [107] (see Figure 1).

### Behavioral tests

#### Spinal reflexes

Thresholds of hindlimb withdrawal reflexes evoked by mechanical stimulation of the knee joint were measured as described in detail previously [106]. Mechanical stimuli of continuously increasing intensity were applied to the knee joint using a calibrated forceps equipped with a force transducer whose output was displayed (in g) on a screen. The area of tissue compressed by the tip of the forceps was 30 mm^2^. Withdrawal threshold was defined as the minimum stimulus intensity that evoked a withdrawal reflex. The test was repeated twice (5 min intervals) and the values were averaged to calculate the threshold (force in g/30 mm^2^).

#### Audible and ultrasonic vocalizations

Vocalizations were recorded and analyzed as described in detail previously [42]. The experimental setup (U.S. Patent 7,213,538) included a custom-designed recording chamber, a condenser microphone (20 Hz to 16 kHz) connected to a preamplifier, an ultrasound detector (25 ± 4 kHz), filter and amplifier (UltraVox 4-channel system; Noldus Information Technology, Leesburg, VA), and data acquisition software (UltraVox 2.0; Noldus Information Technology), which automatically monitored the occurrence of audible and ultrasonic vocalizations within user-defined frequencies and recorded number and duration of digitized events. Vocalizations in the audible and ultrasonic ranges were recorded simultaneously but with different microphones (condenser microphone and ultrasound detector, respectively) connected to separate channels of the amplifier. This computerized recording system was set to ignore sounds outside the defined frequency range. Animals were placed in the recording chamber for acclimation 1 h before the vocalization measurements. The recording chamber ensured the stable positioning of the animal at a fixed distance from the sound detectors and allowed the mechanical stimulation of the knee joint through openings for the hindlimbs. Brief (15 s) innocuous (300 g/30 mm^2^) and noxious (1200 g/30 mm^2^) mechanical stimuli were applied to the knee, using a calibrated forceps (see “Spinal reflexes”). Total duration of vocalizations (arithmetic sum of the duration of individual events) was recorded for 1 min, starting with the onset of the mechanical stimulus. Audible and ultrasonic vocalizations reflect supraspinally organized nocifensive and affective responses to aversive stimuli [106,108].

#### Elevated plus maze test (EPM)

Anxiety-like behavior was determined using the EPM test as described previously [85,106]. The EPM (Columbus Instruments, OH) was constructed from stainless steel to facilitate inter-trial cleaning for elimination of odor cues. A central quadrangle (10 × 10 cm) connected two opposing open arms (50 cm long, 10 cm wide) and two opposing closed arms (50 cm long, 10 cm wide, with 40 cm high walls on both sides), arranged in the shape of a plus. The platform was elevated 70 cm above the floor. An automated photocell system (Multi-Varimex v.1.00; Columbus Instruments, OH, USA) recorded movements of the animal on a personal computer. At the beginning of each trial, the animal was placed onto the central quadrangle facing an open-arm. The EPM was inside a dark enclosure to minimize anxiety levels in the absence of pain. Anxiety-like behavior was determined by measuring the open-arm preference (ratio of open-arm entries to the total number of entries expressed as %) for 5 min. Animals that stayed only in one arm were excluded from experiment.

### Statistical analysis

All averaged values are given as the mean ± standard error of the mean (SEM). GraphPad Prism 3.0 software (Graph-Pad, San Diego, CA) was used for all statistical analysis. For multiple comparisons, one-way analysis of variance (ANOVA) was used followed by Dunnett’s multiple comparisons tests. Statistical significance was accepted at the level P < 0.05.

## Abbreviations

5-HT: Serotonin; BLA: Basolateral nucleus of the amygdala; CeA: Central nucleus of the amygdala; LA: Lateral nucleus of the amygdala; SSRI: Selective serotonin reuptake inhibitor.

## Competing interests

The authors declare that they have no competing interests.

## Authors’contributions

SG and VN conceptualized the hypothesis and designed the study. SG carried out the experiments, analyzed data, prepared figures and wrote the first draft of the manuscript. VN supervised the experiments, directed the data analysis, and finalized the manuscript. All authors read and approved the manuscript.
